# Familial exudative vitreoretinopathy in a 4 generations family of South-East Asian Descendent with *FZD4* mutation (c.1501_1502del)

**DOI:** 10.1186/s40942-022-00384-2

**Published:** 2022-05-16

**Authors:** Yong Zheng Wai, Yong Yuin Chong, Lik Thai Lim, Norhafizah Hamzah, Jamalia Rahmat

**Affiliations:** 1 Ophthalmology Department, Hospital Kuala Lumpur, Kuala Lumpur, Malaysia; 2 Ampang Hospital, Ampang Jaya, Malaysia; 3 Ophthalmology Department, Faculty of Medicine and Health Sciences, Universiti Malaysia Sarawak (UNIMAS), Kota Samarahan, Malaysia; 4 Paediatric Ophthalmology Department, Hospital Tunku Azizah, Kuala Lumpur, Malaysia

**Keywords:** Familial exudative vitreoretinopathy, FEVR, FZD4 genes

## Abstract

**Background:**

Familial Exudative Vitreoretinopathy (FEVR) is a hereditary disorder characterized by peripheral avascular retina with neovascularization. Although FEVR has been thoroughly described in multiple literature publications from different countries, there are currently limited articles describing the phenotypes of FEVR among South-East Asian Descendent. This paper describes the clinical phenotype of the *FZD4* gene with c.1501_1502 deletion in a 4-generation case series of a South East Asian family.

**Methods:**

We reviewed a 4-generation case series of a South-East Asian descendent family consisting of 27 family members with 10 members diagnosed with FEVR. We observed the clinical phenotype of these series of patients, including some of the family members who underwent whole-exome sequencing, PCR amplification and DNA sequencing techniques to identify the mutated gene.

**Results:**

Frameshift mutation (c.1501_1502del) were found in *FZD4* gene in this series of patients with the age ranging from 1 month old to 69 years old. There was a 100% (4/4) of our paediatric patients being diagnosed within 21 days of life. It was also found that 75% of patients (6/8) less than 40 years old exhibited disease asymmetry of 2 stages or more and 80% (8/10) had a history of vitreoretinal surgery or diode laser photocoagulation, with a further 50% of the adult patients identified as legally blind; the mean age of blindness was 18-years-old.

**Conclusions:**

Phenotypic manifestation of *FZD4* gene with c.1501_1502del mutation can be identified within the neonatal period. They have relatively greater clinical asymmetry of 2 stages or more compared to other mutations. Without treatment, most of them will have bilateral severe visual impairment around the adolescent age group.

## Background

Familial exudative vitreoretinopathy (FEVR) was first described by Criswick and Schepens in 1969 as congenital, bilateral vitreoretinopathy with no history of premature birth and oxygen therapy [1]. FEVR is characterized by the peripheral avascular retina and subsequently lead to complication due to retina ischemia [2]. These includes peripheral neovascularization, vitreous haemorrhage, retinal traction with temporal dragging, macular dragging, falciform retinal fold and retinal detachment [2, 3].

FEVR can be inherited in different modes which include autosomal dominant, autosomal recessive and X-linked recessive [4–6]. Autosomal dominant FEVR is the most common, it involves a mutation in *frizzled class receptor 4* (*FZD4*), *low-density lipoprotein receptor protein 5 (LRP5)* or *tetraspanin 12* (*TSPAN12*) [7–9].

*FZD4* genes are located in chromosome 11q14.2 and encode a 7-transmembrane protein of 537 amino acids [10]. FZD4 genes encode Wnt receptor which plays an important role in retinal angiogenesis [9]. To date, there are more than 50 mutations in *FZD4* genes linked with FEVR reported [11]. Deletion of c.1501_1502 is one of the reported mutations in *FZD4* genes. In regards to FZD4 genes related FEVR disease, most of the published articles originate from Caucasian, South Asian (Indian) East Asian (Chinese and Japanese) [9, 11–13]. To our best knowledge, there is currently no published literature describing the phenotypic manifestations of *FZD4* c.1501_1502 deletion among South-East Asian descents.

## Methods

Written informed consent was taken from the patients or their guardians before clinical data and blood samples were collected. This research adhered to the tenets of the Declaration of Helsinki and was registered under the Malaysian National Medical Research Registry (NMRR ID-21-02310-O5A). This study was conducted in Hospital Kuala Lumpur from April 2021 until December 2021.

The diagnosis of FEVR was based on the presence of typical clinical features: peripheral retinal avascular areas or proliferative changes, dragged disc or macula, retinal detachment with exudation or falciform retinal folds and no history of prematurity or oxygen supplementation. We identified one Malaysian patient as a proband with autosomal dominant FEVR and investigated the molecular basis of the disorder with Whole Exome Sequencing (WES). Once the pathogenic gene was identified, some family members who have the phenotype of FEVR went for polymerase chain reaction (PCR) and DNA sequencing techniques for the determination of the targeted mutation. We conducted clinical examination on the other family members and all the clinical data were documented and analysed. Some of the family members were previously treated in our centre, whereby retrospective data extraction was done from the clinical notes.

### Whole exome sequencing (WES)

Whole exome sequencing was performed on genomic DNA using Agilent v6CREv2 targeted sequence capture method to enrich the exome. Direct sequencing of the amplified captured regions was performed using 2 × 100 bp reads on Illumina next-generation sequencing (NGS) systems. Alignments to the human reference genome (hg 19) are performed and annotated variants are identified in the targeted region. Primary data analysis was performed using Illumina DRAGON Bio-IT Platform v.2.03. Secondary and tertiary data analysis was performed using PerkinElmer’s internal ODIN v.1.01 software for single nucleotide variants and Biodiscovery’s NxClinical v.4.3 or Illumina DRAGEN Bio-IT Platform v.2.03 for copy number variation and absence of heterozygosity.

## Results

**Fig. 1:**
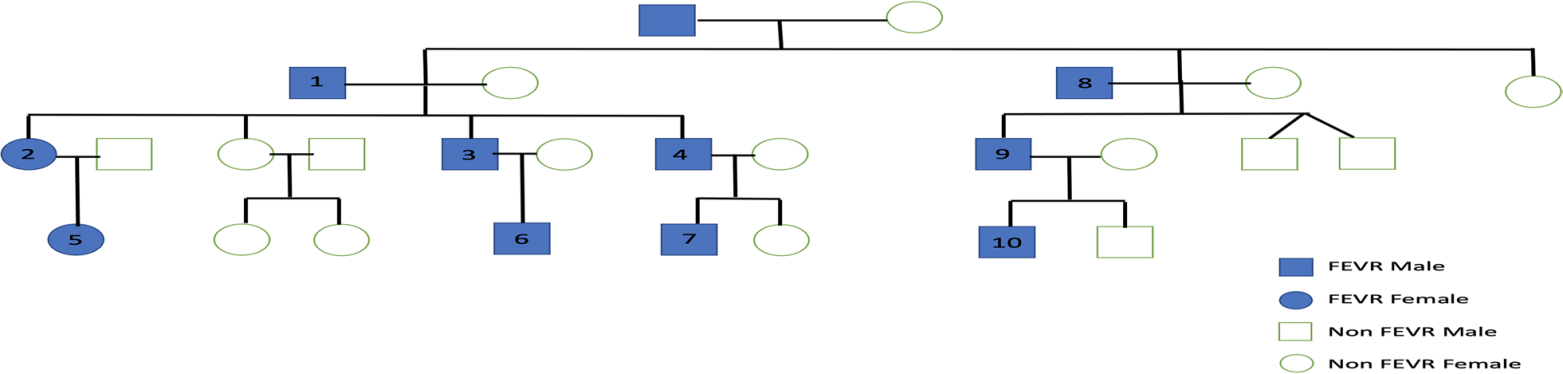
A family pedigree from South-East Asian descent consists of 4 generations, 27 family members with 10 of them diagnosed with FEVR

We identified 10 FEVR patients (20 eyes) within 1 family of South-East Asian descendent. Data were retrospectively extracted and analysed. Of the 10 patients, 7 (70%) were male, their age ranged from 1-month-old to 69 years old. The family pedigree is displayed in Fig. 1.

### Genotype: Frameshift mutation (c.1501_1502del) in *FZD4* gene

Proband is Patient No. 10, WES was done to identify the genetic mutation. Results revealed deletion of two nucleotides at position c.1501_1502 of the *FZD4* gene causing a frameshift in the protein reading frame. PCR amplification and DNA sequencing techniques were performed on Patient No. 9 to detect the c.1501_1502delCT mutation in the *FZD4* gene. Results were positive.

### Phenotype: extremely asymmetric clinical severity

**Fig. 2:**
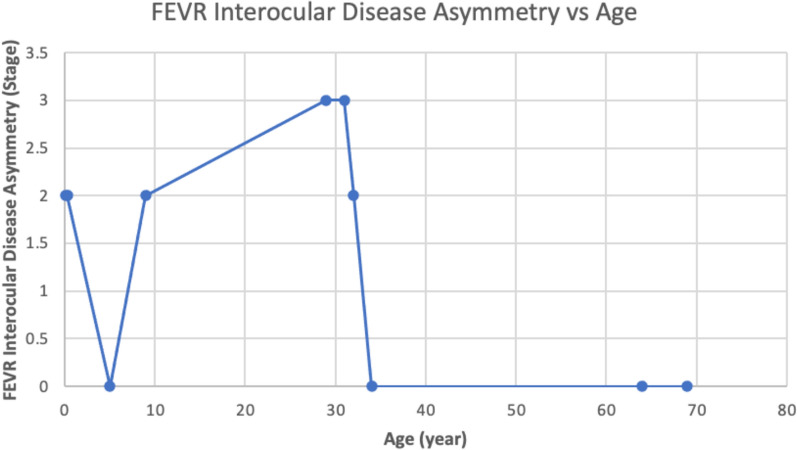
FEVR interocular disease asymmetry vs. age

The clinical severity of FEVR was graded using Revised FEVR Clinical Staging System 2014 [14]. The grading ranged from stage 1 to 5B (Table 1). Among 10 patients, only 4 (40%) have similar staging in both eyes. Clinical asymmetry become more apparent among younger patients, 6 out of 8 patients (75%) that are younger than 40-years-old have asymmetry FEVR clinical grade. All of the patients (100%) that have clinical asymmetry, have differences of at least 2 stages between their eyes (Fig. 2).

**Table 1:** Revised FEVR clinical staging system 2014 and number of eyes

Stage	Description	No. of eyes
1	Avascular periphery or anomalous intraretinal vascularization	
1A	Without exudate or leakage	1
1B	With exudate or leakage	0
2	Avascular retinal periphery with extraretinal vascularization	
2A	Without exudate or leakage	2
2B	With exudate or leakage	1
3	Extramacular retinal detachment	
3A	Without exudate or leakage	2
3B	With exudate or leakage	1
4	Macula-involving retinal detachment, subtotal	
4A	Without exudate or leakage	3
4B	With exudate or leakage	0
5	Total retinal detachment	
5A	Open funnel	0
5B	Closed funnel	10

Visual acuity ranged from logMAR 0.2 to no perception of light. We could not take the visual acuity for the 1-month-old patient. Only 3 out of 9 patients (33.3%) have visual acuity of logMAR 1.0 or better in either eye. All 3 of them had laser therapy or vitreoretinal surgery performed on their better eye.

**Fig. 3:**
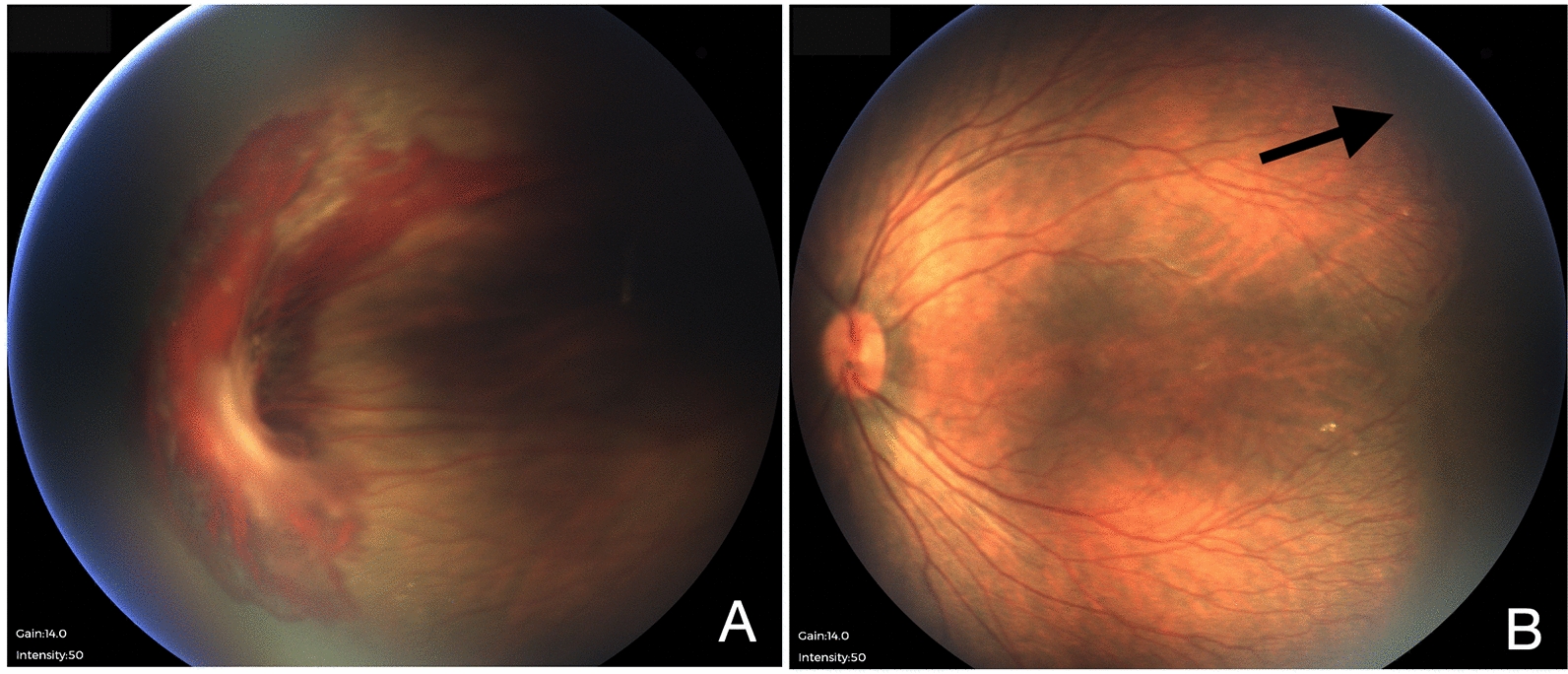
Fundus photo of patient no. 6 shows asymmetric clinical severity. **A ** Right eye shows extramacular full-thickness retina fold with haemorrhage. No obvious exudation was seen. Stage 3A FEVR. **B ** Left eye posterior pole appears flat, arrow shows the border of vascularized retinal with minimal exudates, no laser marks over avascular retina area. Stage 1B FEVR

Among paediatric patients, all 4 patients (No. 5, 6, 7 & 10) were diagnosed in our centre before 21 days of life (Table 2). None of them was born prematurely. All of them had asymmetric retinal folds during our first examination (Fig. 3). The better eye ranged from stage 1 to 4A, the worse eye ranged from 3B to 5B. Diode laser was given to all 4 of them and monitored closely to look for any progression. Despite laser photocoagulation, patient no. 7 still progressed to Stage 4A with retinal fold touching posterior capsule of the crystalline lens. To date, patients 5, 6 & 10 have been clinically stable, but they are too young to conclude that the disease is static.

**Table 2:** FEVR among paediatric age group members

No.	Current age (months)	Age of diagnosis (days-old)	Better eye (stage), VA (LogMAR)	Worse eye (stage), VA (LogMAR)	Procedure	Progression after procedure	Interocular disease asymmetry (stage)
5	1	11	LE 2A*	RE 4A*	BE LIO	Nil	2
6	4	14	LE 1A, 1.74	RE 3B, 2.04	BE LIO	Nil	2
7	60	10	RE 4A, 1.14	LE 4A, 2.04	BE LIO	Progress	0
10	108	21	LE 3A, 0.60	RE 5B, PL	LE LIO RE VR op	Nil	2

*VA* Visual acuity in LogMAR, *PL* Perception of light, *RE* Right eye, *LE *Left eye, *BE* Both eye, *LIO* Diode Laser Indirect Ophthalmoscopy, *VR op* Vitreo-retinal operation, *Nil* No progression after procedure

*Unable to check the visual acuity as patient was too young

The mean age for adult family members is 43.1 years old. (Table 3) 50% (No. 1, 2, 8) of the adult family members are legally blind. All 3 of them are the eldest among all 10 patients. They gave a history of bilateral eye blindness when they were teenagers (ages ranged from 15 to 21 years old). The mean age of their blindness was 18 years old.

**Table 3:** FEVR among adult family members

No.	Current age (years)	Better eye (Stage), VA (LogMAR)	Worse eye (Stage), VA (LogMAR)	Procedure	Progression of disease after procedure	Interocular disease asymmetry (stage)
1	69	RE 5B, NPL*	LE 5B, NPL	LE VR op	Progress	0
2	34	RE 5B, PL	LE 5B, NPL	Nil	-	0
3	31	LE 2A, 0.22	RE 5B, NPL	RE VR op	Nil	3
4	29	LE 2B, 1.30	RE 5B, NPL	LE lens aspiration & IOL Implantation	Nil, Worsening VA due to secondary glaucoma	3
8	64	RE 5B, NPL*	LE 5B, NPL	Nil	-	0
9	32	LE 3A, 0.90	RE 5B, PL	RE VR op	Nil	2

*VA* Visual acuity in LogMAR, *PL* Perception of light, *NPL* No perception of light, *RE,* Right eye, *LE* Left eye, *BE* Both eye, *VR op* Vitreo-retinal operation, *IOL* Intraocular lens, *Nil* None (No procedure done or No progression of disease)

*Both eyes have similar severity

All 6 adult patients complained of unilateral blurring of vision at a very young age (ranged 3 to 10 years old). Subsequently, the better eye will deteriorate and 3 (50%) of them underwent vitreoretinal surgery on their better eye. 1 patient (16.6%) underwent lens extraction over the better eye and suffered from secondary glaucoma, his FEVR remained stable despite absent of vitreoretinal surgery. The remaining 2 adult patients (33.3%) did not seek any medical treatment. The disease remained static after adolescence.

## Discussion

Familial exudative vitreoretinopathy (FEVR, OMIM: 133,780) caused by *FZD4* gene with c.1501_1502 deletion was only briefly described by Toomes et al. [7]. This mutation has been previously reported in individuals with FEVR (PMID: 12172548). There is a paucity of the detailed phenotypic description on c.1501_1502 deletion. To our best knowledge, to date, there has been no article describing the genotypic-phenotypic correlation of this mutation among South-East Asian descents.

### Early detection

*FZD4* gene with c.1501_1502 deletion showed signs of FEVR as early as 10 days of life. Our study managed to diagnose all the 4th generation children during their neonatal period (mean age of diagnosis is 14 days of life) due to positive FEVR family history, compared to a large study that has a mean age of diagnosis of 6 years old.[15] This shows that FEVR caused by *FZD4* gene c.1501_1502delCT mutation can be diagnosed at a very young age within the neonatal period. Screening should be done as early as 10 days old for neonates who have a family history of FEVR, especially for those who had a definitive genetic diagnosis of c.1501_1502 deletion.

### Disease progression and age group

Disease progression was expected to occur before age of 20 years old [16]. Among the 10 patients in our series, 2 of them (No. 1 & 7) exhibited disease progression despite treatment. Progression occurred at the age of 3-years-old (No. 7) and 17-years-old (No. 1). Disease severity remained static for the other 6 patients who received treatment. Only 2 patients did not receive any form of treatment (No. 2 & 8), and both of them translated the natural history of progression in FEVR and became legally blind around adolescent age.

Some studies described FEVR as a lifelong disease where progression can occur at any age after varying periods of apparent quiescent [13, 16]. However, these studies lacked genotypic information. Patients with deletion of c.1501_1502 in *FZD4* gene might have a relatively lesser risk of progression after the adolescent period.

### Disease asymmetry

Interocular clinical asymmetry among FEVR has been largely reported by multiple articles [3, 9, 13, 17]. Generally, 57% of FEVR patients have asymmetry staging, in which 71% presented with the 2 eyes within 1 stage of each other [15]. FEVR with *FDZ4* gene mutation shows 47.4% of asymmetric disease severity which is higher than FEVR patients with *TSPAN12*, *KIF11* or *NDP* mutation [17].

Our results showed greater asymmetry in younger patients with 75% of patients (6/8) who aged younger than 40-years-old exhibited disease asymmetry. All 6 of them (100%) have a difference of 2 stages or more. *Ranchod et al.* reported 71% of FEVR patients have 2 eyes within 1 stage of each other [3]. We hypothesize that the *FZD4* gene c.1501_1502 deletion can produce greater clinical asymmetry compared to other genotype mutations.

Genotype-phenotype association will be the future of diagnostic medicine. FEVR can be caused by varies genotype mutations, and detailed description of the phenotype manifestation, correlating with the genotype mutations for each mutation will help both physicians and patients to understand and manage the disease better, with a more realistic visual prognostic expectations, instead of generalising the disease into a broad spectrum of phenotypic manifestations.

## Conclusions

To our best knowledge, this is the first case series describing the phenotypic expression of *FZD4* gene with c.1501_1502 deletion in South-East Asian descents, spanning a 4-generation case series. Our study highlights that FEVR signs due to c.1501_1502del can appear very early in life (neonatal period) and thus fundus screening is very important among newborns with a family history of FEVR. A deletion in c.1501_1502 could manifest greater clinical asymmetry of 2 stages or more compared to other mutation. Further studies are needed for detail phenotype description in other FEVR genetic mutations.

## Data Availability

All data and materials gathered during this study are included in this study.

## Abbreviations

FEVR: Familial Exudative Vitreoretinopathy
FZD4: Frizzled Class Receptor 4
PCR: Polymerase chain reaction
DNA: deoxyribonucleic acid
LRP5: Low-density lipoprotein receptor protein 5
TSPAN12: Tetraspanin 12
WES: Whole exome sequencing
VA: Visual acuity
RE: Right eye
LE: Left eye
BE: Both eye
PL: Perception of light
NPL: Non perception of light
VR op: Vitreo-retinal operation
IOL: Intraocular lens
KIF11: Kinesin Family Member 11
NDP: Norrin Cystine Knot Growth Factor
